# Supplementary material for: HDAC8-Selective Inhibition by PCI-34051 Enhances the Anticancer Effects of ACY-241 in Ovarian Cancer Cells
Source: Int J Mol Sci. 2022 Aug 3;23(15):8645. doi: 10.3390/ijms23158645 (PMC9369251; doi:10.3390/ijms23158645)
Supplement: Supplementary file 1 [file ijms-23-08645-s001.zip › ijms-1793671-supplementary.pdf]

Kim et al. Figure S1

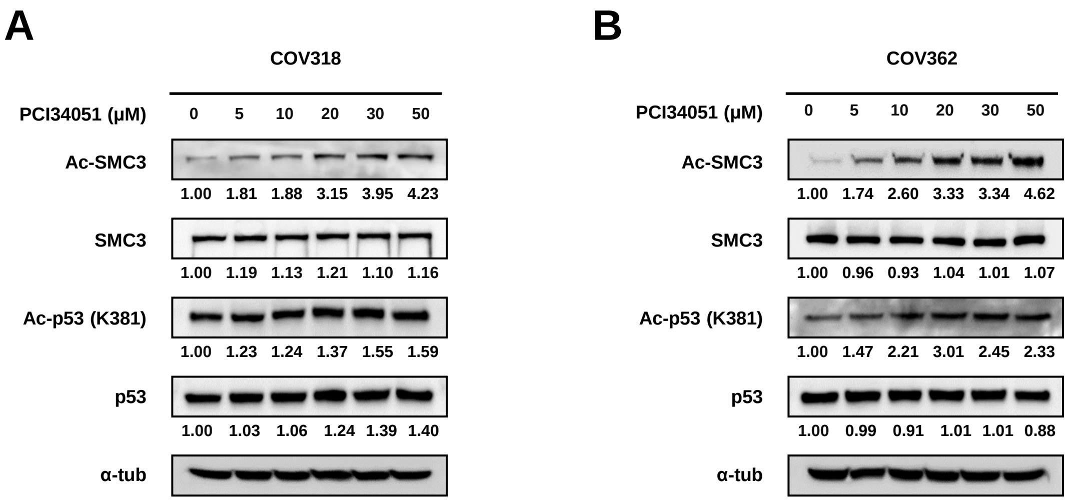

Figure S1. PCI-34051 has limited effects in enhancing p53 stability in p53 mutant ovarian cancer cells. Immunoblotting of acetyl-p53. (**A**) COV318 and (**B**) COV362 cells were treated with indicated doses of PCI-34051 for 24h. Protein expression levels were semi-quantified relative to loading control, α-tubulin.

Kim et al. Figure S2

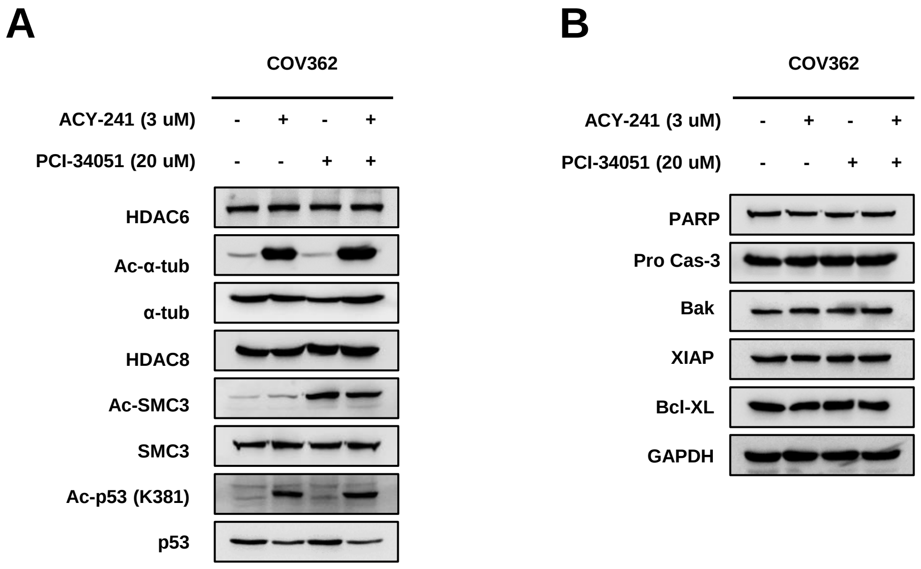

Figure S2. ACY-241 and PCI-34051 does not have a synergistic anti-cancer effect in p53 mutant ovarian cancer cells (A) Immunoblotting of HDAC inhibition markers. Cells were treated with 3 μM ACY-241 and 20 μM PCI-34051 alone or in combination for 24 h. (B) Immunoblotting of pro-apoptotic markers and anti-apoptotic markers in COV362 treated with 3 μM ACY-241 and 20 μM PCI-34051 alone or in combination for 24 h.
